# Real world validation of an AI-based CT hemorrhage detection tool

**DOI:** 10.3389/fneur.2023.1177723

**Published:** 2023-08-03

**Authors:** Dongang Wang, Ruilin Jin, Chun-Chien Shieh, Adrian Y. Ng, Hiep Pham, Tej Dugal, Michael Barnett, Luis Winoto, Chenyu Wang, Yael Barnett

**Affiliations:** ^1^Sydney Neuroimaging Analysis Centre, Camperdown, NSW, Australia; ^2^Brain and Mind Centre, University of Sydney, Sydney, NSW, Australia; ^3^Department of Medical Imaging, St. Vincent’s Hospital, Sydney, NSW, Australia; ^4^Emergency Department, St. Vincent’s Hospital, Sydney, NSW, Australia

**Keywords:** intracranial hemorrhage, clinical validation, AI-based tools, deep learning – artificial intelligence, subarachanoid hemorrhage, post-operative

## Abstract

**Introduction:**

Intracranial hemorrhage (ICH) is a potentially life-threatening medical event that requires expedited diagnosis with computed tomography (CT). Automated medical imaging triaging tools can rapidly bring scans containing critical abnormalities, such as ICH, to the attention of radiologists and clinicians. Here, we retrospectively investigated the real-world performance of VeriScout^™^, an artificial intelligence-based CT hemorrhage detection and triage tool.

**Methods:**

Ground truth for the presence or absence of ICH was iteratively determined by expert consensus in an unselected dataset of 527 consecutively acquired non-contrast head CT scans, which were sub-grouped according to the presence of artefact, post-operative features and referral source. The performance of VeriScout^™^ was compared with the ground truths for all groups.

**Results:**

VeriScout^™^ detected hemorrhage with a sensitivity of 0.92 (CI 0.84–0.96) and a specificity of 0.96 (CI 0.94–0.98) in the global dataset, exceeding the sensitivity of general radiologists (0.88) with only a minor relative decrement in specificity (0.98). Crucially, the AI tool detected 13/14 cases of subarachnoid hemorrhage, a potentially fatal condition that is often missed in emergency department settings. There was no decrement in the performance of VeriScout^™^ in scans containing artefact or postoperative change. Using an integrated informatics platform, VeriScout^™^ was deployed into the existing radiology workflow. Detected hemorrhage cases were flagged in the hospital radiology information system (RIS) and relevant, annotated, preview images made available in the picture archiving and communications system (PACS) within 10 min.

**Conclusion:**

AI-based radiology worklist prioritization for critical abnormalities, such as ICH, may enhance patient care without adding to radiologist or clinician burden.

## Introduction

Intracranial hemorrhage (ICH) is a potentially life-threatening medical event that may occur spontaneously; or in the setting of head trauma or surgical intervention. The etiology of non-traumatic ICH, which has an incidence of >25 per 100,000 person years (1), is diverse and includes hypertension, stroke, ruptured aneurysm, vasculopathy, dural venous sinus thrombosis, arteriovenous fistula, malignancy, anticoagulant use and, rarely, inflammatory disease. Acute ICH is associated with an early mortality of 40%–50% and significant neurological disability in surviving patients (1, 2). ICH subtypes, defined by extravasation of blood into the subdural, extradural, subarachnoid or parenchymal compartments, vary in terms of clinical presentation, imaging features and prognosis. Head computed tomography (CT), the primary para-clinical tool used to investigate patients presenting to emergency departments with headache or focal neurological deficits, facilitates expedited diagnosis and early intervention that may critically determine clinical outcome in ICH (3, 4).

The technical feasibility of artificial intelligence (AI) tools for detecting ICH on head CT has been demonstrated in several recent studies (5–9). However, limited assessment in real-world clinical settings, where patients or scans with characteristics (for example, severe artefacts, postoperative changes, foreign bodies, other brain pathologies) that were not present in the training dataset occur, has shown suboptimal generalizability (10, 11); or performance has been primarily determined by comparison with general, rather than subspecialty expert, radiologist reports (10). Ultimately, patient benefit is also dependent on integration of AI tools with existing radiology workflows, as the use of standalone software interfaces or other additional burdens on clinical staff is a disincentive to their adoption.

We retrospectively investigated the real-world performance of VeriScout^™^, an artificial intelligence (AI) based CT hemorrhage detection and triage tool, in a dataset of unselected non-contrast CT head scans that were consecutively acquired at a large Australian teaching hospital. VeriScout^™^ is designed to triage head CT scans acquired in an outpatient/emergency department setting with a high likelihood of hemorrhage, whether acute or chronic, and flag these scans for expedited reporting through background integration with the Radiology Information System (RIS) and notification in existing clinical systems. VeriScout^™^ is based on a deep learning algorithm and was trained on a dataset of 7,000 expertly labelled head CT scans acquired in an outpatient/emergency department setting. Curation of the training and a separate validation dataset involved the removal of scans with severe metallic or motion artefact; and any post-operative studies. The tool is silently integrated with the existing radiology workflow using an informatics platform (Torana^™^, Sydney Neuroimaging Analysis Centre, Sydney) that provides a binary result (hemorrhage likely/hemorrhage not likely) to the RIS and returns a low resolution (non-diagnostic) bounding box image to the picture archiving and communications system (PACS), providing the reporting radiologist with an indication of the region of the scan that most informed the algorithm’s prediction.

## Methods

The study was approved by the Human Research and Ethics Committee, St. Vincent’s Hospital.

### Dataset

VeriScout^™^ was evaluated retrospectively in an independent dataset of 527 consecutive, unselected non-contrast head CT scans acquired on one of two CT scanners (Philips Ingenuity 2013; Canon Aquilion ONE Prism 2020) with pre-configured parameters (both with tube voltage of 120kVp, tube current 218 mA, slice thickness of 1 mm, reconstructed with soft tissue windows; with convolution kernel as FCXX or UB, and filter type as MEDIUM or UB), at St. Vincent’s Hospital, Sydney. These scanners service the emergency department, outpatients and hospital inpatients. None of the scans acquired for this study were used in the training of the VeriScout^™^ algorithm; and neither scanner was used for the acquisition of any training data. Ground truth for the presence or absence of hemorrhage was iteratively determined by retrospective review of existing radiology reports by a radiology trainee (RJ) with 2 years of specialty experience, followed by secondary, blind review of every scan by a sub-specialty neuroradiologist (YB), who also documented the presence and degree of metal or motion artefact according to the following scale: 0 = no intracranial artefact, 1 = mild artefact with no impact on interpretation, 2 = moderate artefact with potential minor impact on interpretation, 3 = severe artefact with definite impact on interpretation. All intracranial hemorrhage subtypes, including intra-parenchymal, extra-axial (both acute and chronic), subarachnoid and petechial were labelled as positive. Where there was a discrepancy between the report and the neuroradiologist findings, the scan was reviewed by a third radiologist (TD) and a consensus reached by discussion.

### Algorithm and model training

The core algorithm of VeriScout^™^ was developed based on convolutional neural network technology and comprised two independently trained networks based on ResNeXt (12) that provided inference based on the input case as a 3D volume (referred to as the case-level model) and, separately, a group of 2D slices (referred to as the slice-level model). The purpose of the design was to balance the features extracted from the whole case and those from slices. The final inference result was positive (for likely hemorrhage) if either of the predictions is positive.

**Figure 1:**
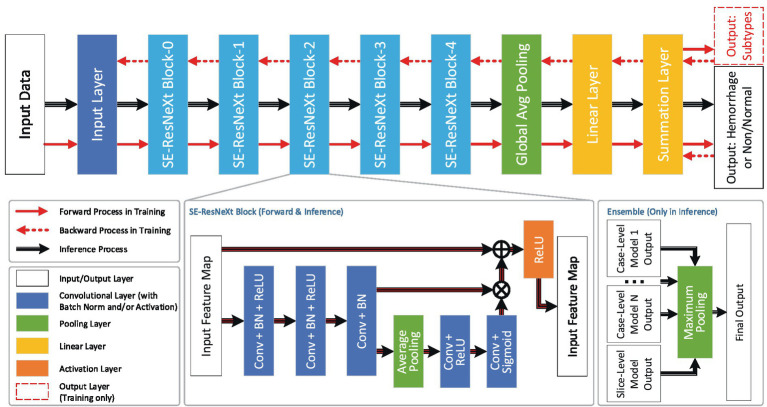
Network structure and data flow. The dataflow and the network structure of VeriScout^™^, in which different layers (including convolutional layers, pooling layers, linear layers and activation layers) are defined and trained based on ResNeXt50 (12). Specifically for the inference step of the device, the results of several case-level models and one slice-level model are fused to generate the final output. See text for more details in training and final output.

Following the network structure and data flow shown in Figure 1, input data was firstly pre-processed with multiple pre-defined grey-level maps (windows) and normalization; output values were then rounded to probabilities and binarized based upon a pre-set threshold that was determined during validation of the algorithm (13).

For the purpose of training neural networks, an extensive brain CT dataset was assembled, consisting of over 20,000 scans sourced from diverse locations throughout Australia. The dataset encompassed more than 50 scanners manufactured by various vendors. Expert clinician reviews of radiology reports were utilized to annotate the dataset, ensuring accurate labeling. To improve the robustness of the network models, data augmentation techniques were employed during the training phase. This involved the random selection of input images from the entire dataset, followed by random cropping, rotation, and contrast adjustments. By employing these techniques, the intention was to encourage the networks to focus on the invariant features present in brain CT images, rather than becoming excessively reliant on features specific to particular scanners or studies.

The networks were optimized to convergence using cross-entropy loss and Adam optimizer based on slice-wise gold labels indicating hemorrhage subtype. Annotations for hemorrhage subtypes were used for training purposes only and the final output was binarized as “hemorrhage likely” or “hemorrhage unlikely.”

### Deployment

**Figure 2:**
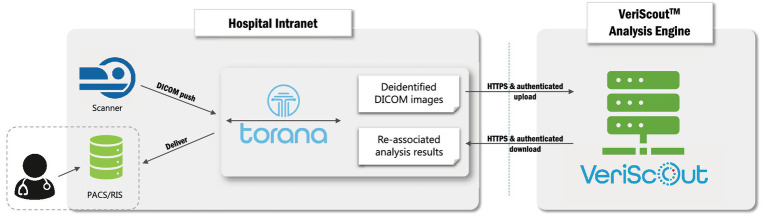
Deployment of hospital-cloud infrastructure. Integration of a hospital-based informatics platform (Torana^™^) with the cloud-based VeriScout^™^ analysis engine. See text for details.

Head CT images were auto-routed from the scanner to a local instance of Torana^™^, a software-based informatics platform that appears as a DICOM node on the local network (Figure 2). On receipt of an appropriate image series (non-contrast head CT), Torana^™^ automatically de-identified the images by replacing all requisite DICOM headers, before securely (HTTPS protocol) routing the scans over the internet to an Amazon Web Services analysis server hosting the containerized VeriScout^™^ analysis algorithm. VeriScout^™^ generated a binary response (hemorrhage/no hemorrhage) and, for positive cases, non-diagnostic labelled DICOM image(s) of the brain slices that most influenced the algorithm’s prediction. This data was automatically routed back to Torana^™^, which reidentified the image(s), transferred these to the appropriate scan session on the hospital PACS and made the result available in the RIS by flagging scans likely to contain hemorrhage with a specific symbol displayed adjacent to the scan ID in the radiologist’s reporting worklist. Additionally, email notifications were sent to the nominated radiologist. All post-acquisition steps described were fully automated, with no input required from end-users or study staff.

### Data analysis

The performance of VeriScout^™^ was assessed by comparison with the final expert consensus ground truth and expressed as sensitivity and specificity (with 95% confidence intervals) and F1 scores. Discrepant results were classed according to the presence or absence of artefact. Additionally, all discrepant DICOM images were visually re-reviewed to determine other potential reasons for failure of the model. The performance of the model was analyzed by referral source; and postoperative scans were also analyzed independently. Any technical issues (such as processing errors) were analyzed for the root cause.

## Results

527 unselected, consecutive CT head scans were acquired, 365 (69.3%) of which were referred from the hospital emergency department (Table 1). Expert consensus found evidence of hemorrhage in 79/527 scans (15.0%), including chronic subdural hematomata and petechial hemorrhage associated with stroke or other pathology such as tumor. The average patient age at the time of scanning was 65.6 years (16.9–97.2). For patients with hemorrhage, the average age was 68.3 years (range 37.7–94.0). There were 45 scans (8.54%) that showed evidence of previous (recent or old) neurosurgery, of which 20 showed evidence of acute or chronic intracranial hemorrhage, predominantly extra-axial in location. When the original report was compared to the multi-radiologist expert consensus, the original report result had a sensitivity of 0.88 and a specificity of 0.98. The sensitivity of the original report was impacted by classification of some subdural collections as hygroma rather than chronic SDH, subacute stroke or tumor associated with petechial hemorrhage as non-hemorrhage and the presence of small amounts of subdural blood associated with recent neurosurgery as post-operative change.

**Table 1:** Clinical features.

Age (range)	65.6y (16.9–97.2)
Gender (M:F)	300:227
Scanner used	
Canon Aquilion ONE	346 (65.7%)
Philips Ingenuity	181 (34.3%)
Referral Source	
Emergency Dept	365 (69.3%)
Other	162 (30.7%)
Predominant Hemorrhage Subtype*	
Intra-parenchymal*	35
Subdural	26
Extradural	0
Subarachnoid	14
Intraventricular	3
Total ICH	78

*Includes chronic subdural hemorrhage and petechial intraparenchymal hemorrhage.

**Figure 3:**
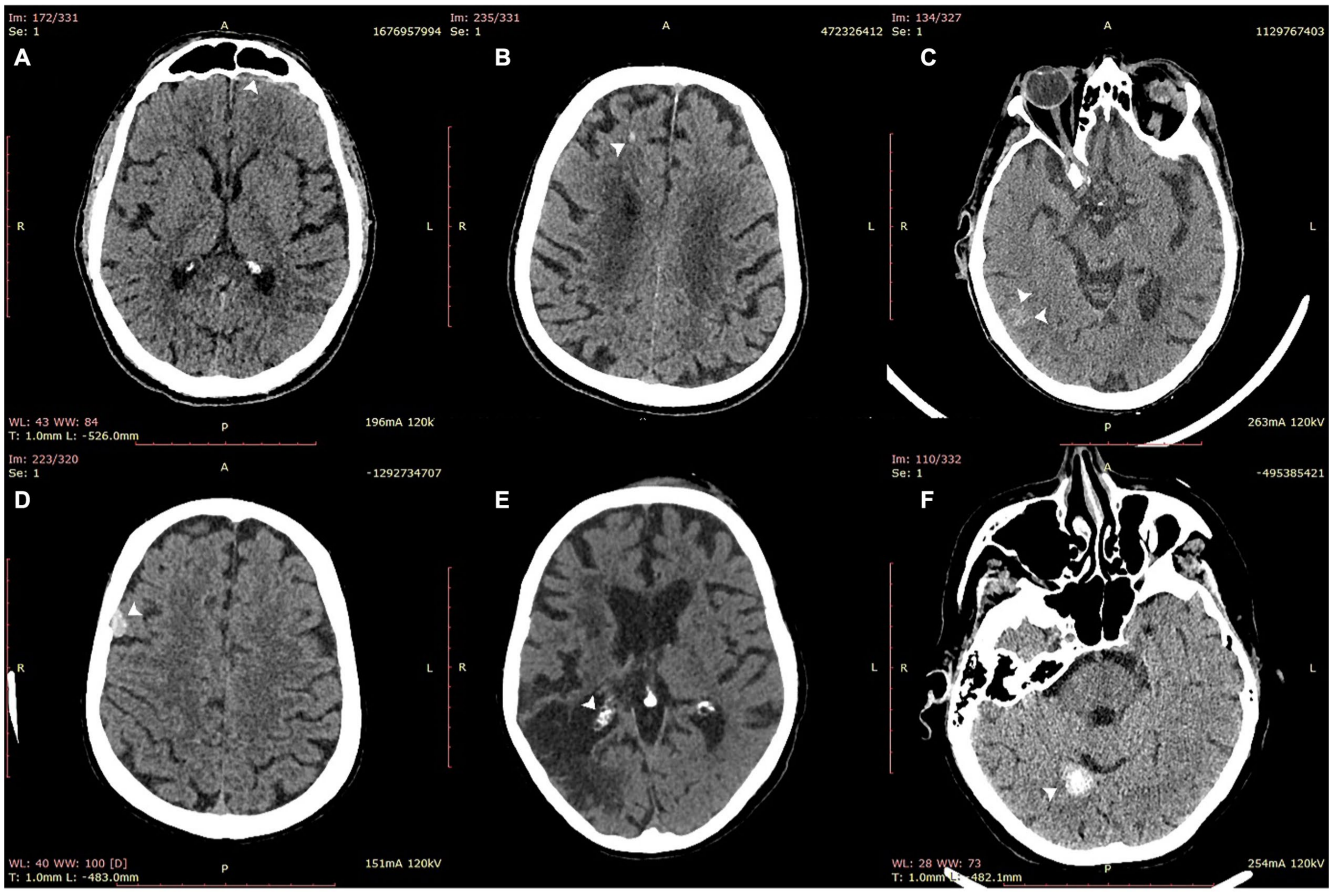
Examples of false negative (top row) and false positive (bottom row) cases misidentified by VeriScout^™^. Upper row: false negative cases of thin subdural hematoma **(A)**; intraparenchymal hemorrhage **(B)**; and focal subarachnoid hemorrhage **(C)**. Bottom row: false positive cases of meningioma **(D)**, choroid plexus calcification adjacent to zone of gliosis from previous stroke **(E)**; and intracranial calcification **(F)**.

For all scans, VeriScout^™^ detected hemorrhage with a sensitivity of 0.92 (CI 0.84–0.96) and a specificity of 0.96 (CI 0.94–0.98) using the expert consensus as ground truth (Table 2), in keeping with the expected performance (namely, sensitivity and specificity of the device should exceed 0.90) of the underlying algorithm (13). Precision and F1 scores are also shown in Table 1. The inclusion of postoperative scans and those with artefacts in our scan cohort did not diminish the overall performance of the algorithm despite their exclusion from the VeriScout^™^ training dataset; and analysis of these scan subsets showed comparable sensitivity and specificity, albeit in limited scan numbers (Table 2). Metallic artefacts primarily affected the posterior fossa structures and temporal lobes, related to dental fillings and metallic earrings/aural implants respectively; and were severe in 18 cases. Severe artefacts related to motion or acquisition issues were present in an additional 4 cases. The algorithm failed to identify hemorrhage (false negative cases) in a small number of cases (*n* = 6: IPH 4, SDH 1, SAH 1, Table 3); examples of these cases are shown in Figure 3, top row. Similarly, there were a number of false positive cases (*n* = 16), accounted for primarily by the presence of intracranial calcification, tumor, severe atrophy (misidentified as chronic subdural hematoma) or, in several cases, no visually identifiable pathology. Examples of false positive cases are shown in Figure 3, bottom row.

**Table 2:** AI-based intracranial hemorrhage detection: outcomes.

	All scans	Referred by Emergency Department	Contains metal artefact (any)	Contains metal artefact (mild or mod)	Postoperative
Positive*	78	31	22	21	20
Negative*	449	334	89	72	25
Total	527	365	111	93	45
Sensitivity (CI)	0.92 (0.84–0.96)	0.90 (0.75–0.96)	0.91 (0.72–0.97)	0.90 (0.71–0.97)	1.00 (0.84–1.00)
Specificity (CI)	0.96 (0.94–0.98)	0.96 (0.93–0.97)	0.98 (0.92–0.99)	1.00 (0.95–1.00)	0.96 (0.80–0.99)
Accuracy	0.96 (0.94–0.97)	0.95 (0.93–0.97)	0.96 (0.91–0.99)	0.98 (0.92–0.99)	0.98 (0.88–1.00)
Precision	0.82 (0.72–0.88)	0.67 (0.52–0.79)	0.91 (0.72–0.97)	1.00 (0.83–1.00)	0.95 (0.77–0.99)
F1 Score	0.87	0.78	0.91	0.95	0.98

*Based on final expert consensus.

**Table 3:** AI system detection rate by hemorrhage subtype.

Hemorrhage subtype	Detection rate
Intra-parenchymal*	0.89 (31/35)
Subdural	0.96 (25/26)
Subarachnoid	0.93 (13/14)
Intraventricular	1.00 (3/3)

*Includes petechial hemorrhage.

**Figure 4:**
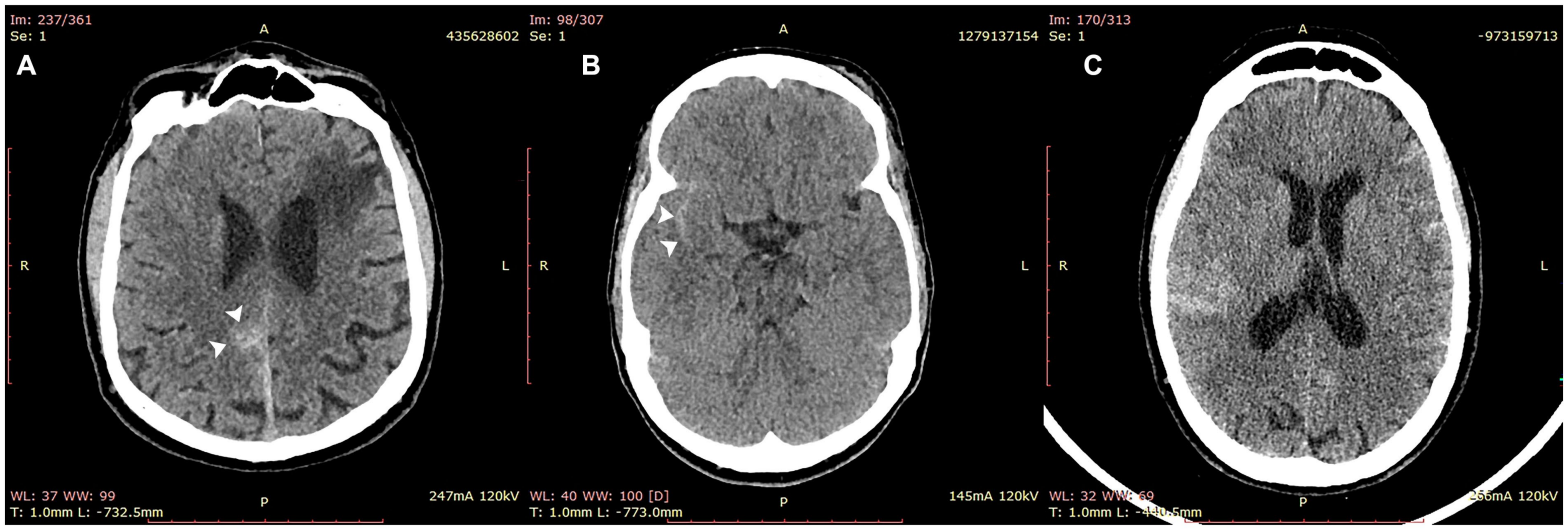
Cases of subarachnoid hemorrhage correctly identified by VeriScout^™^. VeriScout^™^ correctly identified 13/14 cases of subarachnoid hemorrhage (SAH). Examples included cases of focal SAH **(A,B)** and diffuse SAH **(C)**.

The detection rate was excellent across all hemorrhage subtypes and, importantly, was preserved in scans containing SAH (13/14 cases), potentially the most critical hemorrhage subtype (Table 3 and Figure 4). Intraparenchymal hemorrhage, which included petechial hemorrhage, was detected in 31/34 cases (Table 3).

VeriScout^™^ returned a result to the RIS within 10 min in 100% of cases analyzed; and appropriately flagged all positive cases as determined by the algorithm. Upload speed from the hospital network to the cloud analysis server was the primary determinant, with inference completed in less than 1 min in all cases. Integration with the PACS was confirmed by the presence of an appropriate VeriScout^™^ image(s) in the relevant scan session; a technical misconfiguration prevented initial processing in 11 cases, but this was detected in real-time by the informatics platform and all cases were subsequently re-triggered successfully.

## Discussion

### Performance analysis

We demonstrate the performance of an automated AI-based CT head triage system in a hospital radiology department, and its successful integration with our existing PACS-RIS workflow. The sensitivity of VeriScout^™^ for all intracranial hemorrhage subtypes (0.92), including chronic subdural hematomata and petechial hemorrhage, exceeded that of general radiologists (0.88), with only a minor relative decrement in specificity (0.96 vs. 0.98 respectively). Overall discordance between the AI decision and the consensus expert decision was low (4.17%) and comparable with the rate of disagreement between the general radiologist report and the expert decision in both our cohort (3.03%) and previous reports (14–16). Reassuringly, the performance of the AI system was not reduced by the inclusion of postoperative scans and those with severe artefact, despite the exclusion of such scans from the algorithm’s training dataset.

The AI system’s detection rate for SAH (13/14 cases, 0.93), which may require immediate neurosurgical intervention, compared well with the general radiologist report (14/14, 1.00); and significantly exceeds the reported performance of Emergency physicians, who are the first line of defense in many centers with limited radiologist cover after hours (17, 18), when some 80% of SAH cases present (19). In a recent retrospective multicenter study from Kundisch and colleagues, the performance of a commercially available AI algorithm was analyzed on 4,946 scans, showing an “estimated miss rate” for ICH of 12.4%, of which almost 40% were SAH (10). However, only results that were discrepant with the primary radiology report (162/4946) were reviewed by an expert neuroradiologist, a process that resulted in re-classification of >3% of cases in our cohort.

### Features of the tool

There is a clear need for automated medical imaging triaging tools in settings where the volume of imaging studies exceeds the local radiology reporting capacity. A 2015 UK survey indicated that 330,000 patients were waiting more than 1 month for the results of unreported imaging study studies (20), primarily reflecting lack of adequate radiology workforce to meet increasing demand, especially for CT and MRI (21). Separately, advances in imaging technology, which generate increasingly vast amounts of data per acquisition, in the order of thousands of images per CT or magnetic resonance imaging (MRI) scan, contribute to excessive radiologist workload and high rates of burnout in the profession (22). While the emergence of tele-radiology or centralized reporting can ameliorate local radiologist shortages, geographic dissociation from referring clinicians, especially in emergency department settings, increases the risks associated with delayed reporting. Although triaging tools do not address underlying workforce issues, they can critically inform timely management for patients who are potentially most in need of urgent intervention. Due to the retrospective nature of our study, we did not assess the utility of the real-time clinician notification functionality of VeriScout^™^/Torana^™^, which provides an opportunity to further enhance clinical care by expediting appropriate discussion with onsite or offsite radiologists, even prior to scan reporting. Furthermore, our study design precluded a direct assessment of the impact of VeriScout^™^ on patient outcomes; or the perceived benefits to radiologists and referring clinicians.

Explainability, an evolving concept in AI, makes a model’s predictions interpretable and traceable for the end-user; and is particularly important in medical imaging applications (9). By returning non-diagnostic image(s) to the PACS in cases of suspected intracranial hemorrhage, VeriScout^™^ is designed to alert the reporting radiologist to the slices and regions (indicated by a bounding box) that most informed the algorithm’s prediction, a feature that potentially further reduces the time spent reviewing false positive predictions.

Implementation of AI-based triage systems have the capacity to expedite clinical care to patients with critical brain abnormalities by facilitating early reporting and appropriate discussion with referring clinicians. Triage systems, which are not intended to be diagnostic, carry minimal if any risk in the context of algorithm failure, in which instance the workflow simply defaults to standard clinical care. In this study, we retrospectively assessed the performance of VeriScout^™^; however, we also demonstrated near real-time integration with our existing RIS-PACS workflow through an associated informatics platform, Torana^™^; and therefore the feasibility of full deployment in a clinical setting.

Data privacy concerns are a roadblock to the implementation of imaging AI in some jurisdictions and centers (23). Our head CT scans were processed by a secure cloud (Amazon Web Services) analysis server following on-site, automated deidentification of DICOM headers; additionally, image transfer to the server used a secure protocol (HTTPS); and off-site data was automatically deleted when processing completed. While on-site solutions obviate privacy and other governance concerns, these are not scalable and are more difficult to maintain and update. Stringent compliance with HIPAA, GDPR or other relevant regulations; and use of vendor systems that are compliant with international (such as ISO 13485) and local medical device regulations substantially reduces the risk of exposure of protected health information. Additional precautions, such as ‘defacing’ software that automatically removes pixels that could be feasibly reconstructed and visually identified from 3D datasets (23), may become compulsory for applications that require off-site data processing.

### Future work

VeriScout^™^ is designed to detect a high likelihood of hemorrhage of any type or extent, and triage these cases for expedited clinical review. We recognize that, in some cases, very small amounts of subarachnoid or subdural blood, particularly in the setting of trauma, may have little or no clinical significance in terms of patient management. Conversely, the detection of small amounts of non-traumatic subarachnoid blood may critically impact patient management. Referral information is extremely important in determining the clinical significance of CT findings, including small amounts of ICH. Unfortunately, no referral information was available in the current study; however, the AI tool missed only 6 hemorrhages, only one of which (focal SAH, see Figure 3C) was deemed likely to be of clinical significance by an expert neuroradiologist (YB). As VeriScout^™^ is not designed as a diagnostic tool, and even small hemorrhages may influence patient management (for example, the administration of antiplatelet or anticoagulant therapy), we believe that our algorithm appropriately triages all hemorrhages for expedited human interpretation in the appropriate clinical context. Future, more general AI algorithms, which are able to access and integrate clinical and imaging information, will likely further refine worklist prioritization and, potentially, diagnostic use cases.

AI is likely to transform medical imaging over the coming decades. Worklist prioritization for critical abnormalities, as described here, is one application of medical imaging AI that enhances patient care without adding to radiologist/clinician burden. Increasing demand for the quantitative monitoring of chronic disease, particularly in neurology (24), is a further application that value-adds to qualitative reporting and has the potential to facilitate precision treatment strategy in individual patients. Informatics tools that seamlessly integrate these tools into existing clinical workflows will critically determine their adoption. Finally, the rapidity with which AI-based imaging tools are being developed mandates the implementation of national and local governance frameworks to facilitate their safe adoption in clinical practice.

## Data availability statement

The original contributions presented in the study are included in the article/supplementary material, further inquiries can be directed to the corresponding author.

## Ethics statement

The studies involving human participants were reviewed and approved by Human Research and Ethics Committee, St. Vincent’s Hospital. The patients/participants provided their written informed consent to participate in this study.

## Author contributions

The project was adminstrated and supervised by CW and YB, and conceptualized by MB, CW, YB, and DW. Methodology was developed by MB, DW, and RJ. Data was collected, analyzed and validated by RJ and DW, and further reviewed by MB, YB, and TD. The algorithm and its clinical integration were developed by DW and CS; and deployed in the clinical workflow by CS, AN, HP, and LW. The paper was initially drafted by DW and MB. All authors contributed to the article and approved the submitted version.

## Funding

This study is funded by Cooperative Research Centres Projects (CRCPFIVE000141), Medical Research Future Fund (MRFFAI000085) and the Research Training Program of Australian Government.

## Conflict of interest

DW, CS and CW are part-time employees at the Sydney Neuroimaging Analysis Centre (SNAC). MB has received institutional support for research, speaking and/or participation in advisory boards for Biogen, Merck, Novartis, Roche, and Sanofi Genzyme, and is a research consultant to RxPx and research director for the SNAC. YB and TD are consulting radiologists for SNAC.

The remaining authors declare that the research was conducted in the absence of any commercial or financial relationships that could be construed as a potential conflict of interest.

## Publisher’s note

All claims expressed in this article are solely those of the authors and do not necessarily represent those of their affiliated organizations, or those of the publisher, the editors and the reviewers. Any product that may be evaluated in this article, or claim that may be made by its manufacturer, is not guaranteed or endorsed by the publisher.

## References

[1] Li X Zhang L Wolfe CDA Wang Y. Incidence and long-term survival of spontaneous intracerebral hemorrhage over time: a systematic review and meta-analysis. Front Neurol. (2022) 13:819737. doi: 10.3389/fneur.2022.819737, PMID: 35359654 PMC8960718

[2] van Asch CJ Luitse MJ Rinkel GJ van der Tweel I Algra A Klijn CJ. Incidence, case fatality, and functional outcome of intracerebral hemorrhage over time, according to age, sex, and ethnic origin: a systematic review and meta-analysis. Lancet Neurol. (2010) 9:167–76. doi: 10.1016/S1474-4422(09)70340-0, PMID: 20056489

[3] Hostettler IC Seiffge DJ Werring DJ. Intracerebral hemorrhage: an update on diagnosis and treatment. Expert Rev Neurother. (2019) 19:679–94. doi: 10.1080/14737175.2019.1623671 31188036

[4] Cordonnier C Demchuk A Ziai W Anderson CS. Intracerebral hemorrhage: current approaches to acute management. Lancet. (2018) 392:1257–68. doi: 10.1016/S0140-6736(18)31878-6 30319113

[5] Ye H Gao F Yin Y Guo D Zhao P Lu Y. Precise diagnosis of intracranial hemorrhage and subtypes using a three-dimensional joint convolutional and recurrent neural network. Eur Radiol. (2019) 29:6191–201. doi: 10.1007/s00330-019-06163-2, PMID: 31041565 PMC6795911

[6] Rava RA Seymour SE LaQue ME Peterson BA Snyder KV Mokin M. Assessment of an artificial intelligence algorithm for detection of intracranial hemorrhage. World Neurosurg. (2021) 150:e209–17. doi: 10.1016/j.wneu.2021.02.134 33684578

[7] Heit JJ Coelho H Lima FO Granja M Aghaebrahim A Hanel R. Automated cerebral hemorrhage detection using RAPID. AJNR Am J Neuroradiol. (2021) 42:273–8. doi: 10.3174/ajnr.A6926, PMID: 33361378 PMC7872180

[8] Kuo W Hne C Mukherjee P Malik J Yuh EL. Expert-level detection of acute intracranial hemorrhage on head computed tomography using deep learning. Proc Natl Acad Sci U S A. (2019) 116:22737–45. doi: 10.1073/pnas.1908021116, PMID: 31636195 PMC6842581

[9] Wang X Shen T Yang S Lan J Xu Y Wang M. A deep learning algorithm for automatic detection and classification of acute intracranial hemorrhages in head CT scans. Neuroimage Clin. (2021) 32:102785. doi: 10.1016/j.nicl.2021.102785, PMID: 34411910 PMC8377493

[10] Kundisch A Honning A Mutze S Kreissl L Spohn F. Deep learning algorithm in detecting intracranial hemorrhages on emergency computed tomographies. PLoS One. (2021) 16:e0260560. doi: 10.1371/journal.pone.0260560, PMID: 34843559 PMC8629230

[11] Ginat DT. Analysis of head CT scans flagged by deep learning software for acute intracranial hemorrhage. Neuroradiology. (2020) 62:335–40. doi: 10.1007/s00234-019-02330-w 31828361

[12] Xie S Girshick R Dollár P Tu Z He K: Aggregated residual transformations for deep neural networks. 2017 IEEE conference on computer vision and pattern recognition (CVPR), 21–26: (2017), 5987–5995.

[13] Wang D Wang C Masters L Barnett M. Masked multi-task network for case-level intracranial hemorrhage classification in brain ct volumes. International Conference on Medical Image Computing and Computer-Assisted Intervention. Springer International Publishing, (2020) 145–154.

[14] Roszler MH McCarroll KA Rashid T Donovan KR Kling GA. Resident interpretation of emergency computed tomographic scans. Investig Radiol. (1991) 26:374–6. doi: 10.1097/00004424-199104000-00016 2032826

[15] Erly WK Berger WG Krupinski E Seeger JF Guisto JA. Radiology resident evaluation of head CT scan orders in the emergency department. AJNR Am J Neuroradiol. (2002) 23:103–7. PMID: 11827881 PMC7975501

[16] Erly WK Ashdown BC Lucio RW Carmody RF Seeger JF Alcala JN. Evaluation of emergency CT scans of the head: is there a community standard? AJR Am J Roentgenol. (2003) 180:1727–30. doi: 10.2214/ajr.180.6.1801727 12760951

[17] Austin R Price J Boyle A. Can emergency physicians accurately interpret computed tomography scans performed for suspected nontraumatic subarachnoid hemorrhage: a cross-sectional study. Eur J Emerg Med. (2018) 25:447–8. doi: 10.1097/MEJ.0000000000000560, PMID: 30379717

[18] Spitler K Vijayasarathi A Salehi B Dua S Azizyan A Cekic M. 24/7/365 neuroradiologist coverage improves resident perception of educational experience, referring physician satisfaction, and turnaround time. Curr Probl Diagn Radiol. (2020) 49:168–72. doi: 10.1067/j.cpradiol.2018.09.004, PMID: 30391225

[19] Stewart H Reuben A McDonald J. LP or not LP, that is the question: gold standard or unnecessary procedure in subarachnoid hemorrhage? Emerg Med J. (2014) 31:720–3. doi: 10.1136/emermed-2013-202573 23756363

[20] Royal College of Radiologists. Unreported X-rays, computed tomography (CT) and magnetic resonance imaging (MRI) scans: results of a snapshot survey of English National Health Service (NHS) trusts. London: Royal College of Radiologists (2015).

[21] McDonald RJ Schwartz KM Eckel LJ Diehn FE Hunt CH Bartholmai BJ. The effects of changes in utilization and technological advancements of cross-sectional imaging on radiologist workload. Acad Radiol. (2015) 22:1191–8. doi: 10.1016/j.acra.2015.05.007, PMID: 26210525

[22] Stephenson N. 2016 RANZCR clinical radiology workforce census report: Australia. The Royal Australia and New Zealand College of Radiologists (2018).

[23] Lotan E Tschider C Sodickson DK Caplan AL Bruno M Zhang B. Medical imaging and privacy in the era of artificial intelligence: myth, fallacy, and the future. J Am Coll Radiol. (2020) 17:1159–62. doi: 10.1016/j.jacr.2020.04.007, PMID: 32360449 PMC7484310

[24] Goodkin O Pemberton H Vos SB Prados F Sudre CH Moggridge J. The quantitative neuroradiology initiative framework: application to dementia. Br J Radiol. (2019) 92:20190365. doi: 10.1259/bjr.20190365, PMID: 31368776 PMC6732931

